# Liquor Flavour Is Associated With the Physicochemical Property and Microbial Diversity of Fermented Grains in Waxy and Non-waxy Sorghum (*Sorghum bicolor*) During Fermentation

**DOI:** 10.3389/fmicb.2021.618458

**Published:** 2021-06-17

**Authors:** Chunjuan Liu, Xiangwei Gong, Guan Zhao, Maw Ni Soe Htet, Zhiyong Jia, Zongke Yan, Lili Liu, Qinghua Zhai, Ting Huang, Xiping Deng, Baili Feng

**Affiliations:** ^1^College of Life Sciences, Northwest A&F University, Yangling, China; ^2^College of Agronomy, State Key Laboratory of Crop Stress Biology in Arid Areas/Northwest A&F University, Yangling, China; ^3^Shaanxi Xifeng Liquor Co., Ltd., Baoji, China

**Keywords:** alcohols, bacteria, esters, fungi, physicochemical parameters, Xifeng liquor

## Abstract

The fermentation process of Chinese Xifeng liquor involves numerous microbes. However, the sources of microbes in fermented grain and the link between liquor flavour and physicochemical properties and microbial diversity during fermentation still remain unknown. Herein, two waxy (JiNiang 2 [JN-2] and JinNuo 3 [JN-3]) and four non-waxy (JiZa 127 [JZ-127], JinZa 34 [JZ-34], LiaoZa 19 [LZ-19], and JiaXian [JX]) sorghum varieties were selected for the comprehensive analysis of the relationship between liquor flavour and the physicochemical properties and microbial diversity of fermented grains. Results showed that ethyl acetate was the main flavour component of JZ-127, JZ-34, and JX, whereas ethyl lactate was mainly detected in JN-2, JN-3, and LZ-19. Ethyl lactate accounted for half of the ethyl acetate content, and JX exhibited a higher liquor yield than the other sorghum varieties. The fermented grains of waxy sorghum presented higher temperature and reducing sugar contents but lower moisture and starch contents than their non-waxy counterparts during fermentation. We selected JN-3 and JX sorghum varieties to further investigate the microbial changes in the fermented grains. The bacterial diversity gradually reduced, whereas the fungal diversity showed nearly no change in either JN-3 or JX. *Lactobacillus* was the most abundant bacterial genus, and its level rapidly increased during fermentation. The abundance of *Lactobacillus* accounted for the total proportion of bacteria in JX, and it was higher than that in JN-3. *Saccharomyces* was the most abundant fungal genus in JX, but its abundance accounted for a small proportion of fungi in JN-3. Four esters and five alcohols were significantly positively related to Proteobacteria, Bacteroidetes, and Actinobacteria; Alphaproteobacteria, Actinobacteria, and Bacteroidia; Bacillales, Bacteroidales, and Rhodospirillales; and *Acetobacter*, *Pediococcus*, and *Prevotella_7*. This positive relation is in contrast with that observed for Firmicutes, Bacilli, Lactobacillales, and *Lactobacillus*. Meanwhile, *Aspergillus* was the only fungal microorganism that showed a significantly negative relation with such compounds (except for butanol and isopentanol). These findings will help in understanding the fermentation mechanism and flavour formation of fermented Xifeng liquor.

## Introduction

Sorghum (*Sorghum bicolor* L. Moench) is a widely planted cereal crop found in semiarid regions because of its strong resistance to drought, saline and poor soil conditions. This plant has high nutrient contents, such as phenolic antioxidant compounds, and can be used in livestock feed and biofuel production (Ananda et al., 2011; Xu et al., 2018). Despite the reduced sorghum plantation area in China, sorghum is widely adopted as a raw material for brewing in the wine industry (Tawaba et al., 2013). On the basis of usage, sorghum is divided into four types, namely, grain, sweet, grass and broom sorghum (Lu and Dahlberg, 2001). Brewing liquor, such as Moutai, Wuliangye, Luzhoulaojiao, and Langjiu, and vinegar is based on sorghum grains as raw materials. Grass sorghum is divided into waxy and non-waxy types based on the ratio of amylose and amylopectin (Sang et al., 2008). The selection of raw materials may influence the liquor’s quality and value. Therefore, generalised plantation and application of different sorghum varieties are important for the development of the sorghum industry (Elhassan et al., 2015).

Chinese liquor is a famous traditional Chinese drink. It is divided into five main categories, namely, strong aroma, light aroma, soy sauce aroma, sweet honey and miscellaneous type liquors (Fan and Qian, 2006). Although alcohol and water account for 97–98% of liquor, other micro-components determine its flavour and quality (Han et al., 2018). The flavour of Chinese liquor is enhanced by aroma compounds, including esters, alcohols, acids, aldehydes, ketones, acetals, and heterocyclic compounds. Oswald (2003) observed that esters, especially ethyl octanoate and ethyl decanoate, and alcohols are amongst the key aroma compounds formed at the end of winemaking. Aroma compounds have been detected in Chinese liquor. Ethyl hexanoate is believed to be a primary aroma compound in strong aroma-type Chinese liquor (Shen, 1996). By comparing the volatile compounds of Wuliangye with those of Moutai, Kim et al. (2009) found that diethyl succinate only exists in Wuliangye. Therefore, liquor aroma compounds exist in various proportions to create different liquor flavours and types and thus can be used to improve the quality of liquor production.

The quality of liquor made from sorghum is related to the sorghum material, local climate, brewing technology and microbial population (He et al., 2019). The structure and metabolic activities of the microbial community are key factors that determine liquor quality. Microorganisms native to the domain Bacteria and those affiliated with Eukaryota play a crucial role in Chinese liquor fermentation (Wang P. et al., 2017). Jin et al. (2019) reported that Bacillales, Enterobacteriales, and Lactobacillales are dominant bacteria, whereas *Candida*, *Trichoderma*, *Aspergillus*, *Trichosporon*, and *Thermomyces* are predominant fungal communities in Moutai Daqu. Wang C. et al. (2008) discovered different microorganisms in various categories of Chinese liquor and reported that Bacilli, Bacteroidetes and Clostridia are predominant in strong aroma type fermented grains, whereas Bacilli, Flavobacteria, and Gammaproteobacteria are predominant in fermented grains with roasted sesame aroma type. Lactic acid bacteria are also important microbial groups in the Chinese liquor brewing industry; they are used to improve the flavour of liquors (Fang et al., 2015). The organic acids produced by the metabolism of lactic acid bacteria are the main factor affecting the acidity of liquors; these compounds benefit starch saccharification and ethanol fermentation by maintaining the acidity of the brewing environment (Wang et al., 2014). The predominant key microorganisms in fermented grains and the effect of their metabolic products are crucial for the development of liquor flavour. Therefore, analysing the changes in the microbial community structure of fermented grains is important to reveal the mechanism of flavour production, control fermentation and ensure the stability of liquor quality (Cocolin et al., 2000).

Liquors are complex mixtures consisting of hundreds of component substances present in different concentrations (Fan et al., 2012). With the rapid development of sequencing technology, the relationship between microbial community structure and liquor quality has become a research focus (Zheng et al., 2012; Kim et al., 2018). However, limited studies have focused on raw materials that cause differences in the brewing environment (Wu et al., 2015). The mechanism by which microbes change during fermentation and how they affect the flavour of liquors remain unclear. Therefore, this study aimed to (1) examine the changes in the physicochemical parameters of sorghum varieties during fermentation; (2) analyse the microbial (bacterial and fungal) diversity and composition with Illumina sequencing of the 16S rRNA gene and internal transcribed spacer (ITS) gene; (3) determine the volatile compounds in alcohol by liquid-liquid microextraction (LLME) with gas chromatography-mass spectrometry (GC-MS); and (4) evaluate the relationships amongst the physicochemical parameters, microbial community and liquor flavour. Different sorghum varieties were systematically compared to analyse the comprehensive effects of physicochemical parameters and microbial community changes on liquor quality during liquor fermentation and to ascertain the sorghum varieties suitable for brewing liquor. Furthermore, gaining insights into the parameter changes during liquor fermentation and the development of beneficial bacteria are important for ensuring liquor quality.

## Materials and Methods

### Materials and Sample Collection

Six sorghum varieties, namely, JiNiang 2 (JN-2), JinNuo 3 (JN-3), JiZa 127 (JZ-127), JinZa 34 (JZ-34), LiaoZa 19 (LZ-19), and JiaXian sorghum (JX), were used (Supplementary Figure 1). These varieties were divided into two genotypes. JN-2 and JN-3 are waxy sorghums with low amylose contents (not exceeding 10%) (Yang et al., 2018). JZ-127, JZ-34, LZ-19, and JX are non-waxy sorghums with high amylose contents. Supplementary Table 1 shows the quality properties, including starch, protein and fat contents, of the six sorghum grain varieties.

In May 2019, sampling was conducted during fermentation at Xifeng Liquor Limited Liability Company in Fengxiang Town, Shaanxi Province, China (N34°26′6.74″, E107°29′52.51″). Three paralleled fermentation pits were selected for fermented grain sampling. The fermented grain samples were collected with a special sampler (a tweezer-like tool with spoon-like heads and sharp edges) from the middle of the pits and the upper, middle and lower parts of the four pit corners. The samples were then mixed to form one sample. The fermented grain samples were collected after fermentation for 1, 3, 6, 9, 12, 15, 18, 21, 24, and 26 days. Finally, the samples were transported to the laboratory on ice and kept at −80°C.

### Measurement of Liquor Quality and Yield

#### Liquid-Liquid Microextraction-Gas Chromatography-Mass Spectrometry

Exactly 18 mL of 10% (*v*/*v*) finished alcohol sample was aspirated and added with 6 g of sodium chloride until saturation, followed by the addition of 6 μL of 5.85 mg/100 mL butyl hexanoate as the internal standard, 1 mL of re-distilled ether with shaking for 3 min and 1 μL of organic phase after standing and stratification to perform GC-MS analysis. Compound concentrations were calculated based on the ratio of the peak area of a compound relative to the peak area of the internal standard based on the calibration curves.

#### Gas Chromatography-Mass Spectrometry Analysis Conditions

The inlet temperature was 250°C, the carrier gas was high-purity helium (purity 99.999%), and the column flow rate was 2 mL/min with splitless injection. The program temperature conditions were as follows: holding at 50°C for 2 min, 6°C/min rate to 230°C and holding for 15 min. The MS conditions were as follows: electron ionisation source, ionisation voltage of 70 eV, ion source temperature of 230°C and spectrum scan range of 35–350 amu.

#### Liquor Yield

The liquor was distilled from the fermented grains obtained from the pit, the comprehensive alcohol content of the original wine was calculated at 65° (Zhou et al., 2015), and the liquor yield was calculated at a specified ratio of grain (900 kg) input to liquor output.

Liquor yield (%) = alcohol output/grain input × 100%.

#### Analysis of the Physicochemical Properties of Fermented Grains

The moisture content of fermented grains was determined by quarter sampling, which involved weighing 10 g of fermented grain samples and placing them in a petri dish for flattening. The samples were then baked in an infrared oven for approximately 20 min and then weighed after cooling to room temperature. The moisture content was calculated using the equation from the work of Wang P. et al. (2017):

$$M\,o\,i\,s\,t\,u\,r\,e=\frac{W-W\,0}{W}\times 100\%$$

where *W* is the initial sample weight and *W*_0_ is the sample weight after drying.

Exactly 5 g of fermented grain samples was weighed and added with 100 mL of 1:4 hydrochloric acid solution to determine the starch and reducing sugars. The bottle was pressed on a reflux condenser, hydrolysed in boiling constant-temperature water bath for 30–60 min, removed from the bath, rapidly cooled and neutralised with sodium hydroxide. The filtrate was collected in a 500 mL volumetric flask after filtering with an absorbent cotton. The residue was washed thoroughly with water to a constant volume of 500 mL. Exactly 5 mL each of Feilin A and B solutions was pipetted to a 150 mL flask, added with 9 mL of 0.1% standard glucose solution from the burette and shaken well. The sample was then heated on an electric stove until boiling and titrated with 0.1% standard glucose solution until the blue colour disappeared immediately, and the solution turned to light yellow. The specific steps were described by Chen et al. (2013).

The acidity of fermented grains was measured by weighing 10 g of fermented grain samples, adding 50 mL of distilled water with stirring and soaking for 30 min in a triangle bottle. After filtering with a filter paper, 5 mL of the supernatant was transferred to a triangular bottle and added with 25 mL of distilled water and two drops of phenolphthalein indicator with stirring. Then, the solution was titrated with 0.1 M sodium hydroxide standard solution until it became reddish. The volume of sodium hydroxide standard solution was recorded to calculate the acidity of the fermented grains (Wang P. et al., 2017).

A total of 100 g of fermented grain samples was weighed and added with 200 mL of distilled water in a 500 mL distillation flask. Then, the mixture was added with 100 mL of effluent in a graduated cylinder to determine the alcohol content of the fermented grains. The distilled liquor was mixed evenly and placed gently on an alcohol meter in a graduated cylinder, and the alcohol degree was corrected to a value of 20°C. After stabilisation, the alcohol degree was read based on the alcohol and temperature correction table.

#### Fermented Grain DNA Extraction, Polymerase Chain Reaction Amplification, and Illumina Sequencing

The fermented grains of JN-3 and JX sorghum were used for microbial composition analysis. Samples were obtained on days 3, 9, 15, and 26 after fermentation. Microbial DNA was extracted using a PowerSoil DNA Isolation Kit (MoBio Laboratories, Carlsbad, CA, United States) following the manual. The purity and quality of the genomic DNA were checked on 0.8% agarose gels. Primers 16S-F (ACTCCTACGGGAGGCAGCAG) and 16S-R (GGACTACHVGGGTWTCTAAT) were used to amplify the bacterial 16S gene for the V3-V4 hypervariable regions. Primers ITS-F (5′-CTTGGTCATTTAGAGGAAGTAA-3′) and ITS-R (5′-TGCGTTCTTCATCGATGC-3′) were used to amplify the fungal ITS region. The ultra-PAGE purified primers were bought from Invitrogen, China. The polymerase chain reaction (PCR) products were purified using an Agencourt AMPure XP Kit. Deep sequencing was performed on MiSeq platform at Allwegene Company (Beijing). The sequence data associated with this project have been deposited at the National Center for Biotechnology Information (accession numbers: PRJNA670598 for bacteria and PRJNA670601 for fungi).

#### Processing of 16S rRNA and ITS Gene Data

The raw sequences of bacterial and fungal reads were initially trimmed using Mothur, and sequences satisfying the following criteria were considered: (1) precise primers and barcodes, (2) quality score > 30, and (3) length > 200 bp. The Ribosomal Database Project classifier tool was used to classify all sequences into different taxonomic groups (Wang et al., 2007). The qualified reads were separated using sample-specific barcode sequences and trimmed with Illumina Analysis Pipeline version 2.6. Then, the dataset was analysed using QIIME. The sequences were clustered into operational taxonomic units (OTUs) at 97% similarity level to generate rarefaction curves and calculate the richness and diversity indices (Cole et al., 2014).

### Statistical Analyses

Taxonomic alpha diversity was calculated as the estimated community diversity by Shannon index using the Mothur software package (v.1.30.1). Non-metric multidimensional scaling (NMDS) was utilised to evaluate the ecological distances of different samples based on the weighted UniFrac distances via EMPeror, and changes in the microbial structure during the fermentation period were considered to indicate the microbial beta diversity. The relationships between the physicochemical properties of fermented grains and liquor quality were determined by Spearman’s correlation analysis (SPSS 19.0, SPSS Inc., Chicago, United States). To determine and visualise the correlations, we created networks by using Gephi (Web Atlas, Paris, France) (Bastian et al., 2009). Each node represents a genus, whereas each edge indicates a strong and significant correlation between nodes. All analyses were carried out in R environment with VEGAN and Hmisc (Vanderbilt University, Nashville, TN, United States) packages (Harrell, 2010).

The data, including the physicochemical properties and microbial communities in the fermented grains, were analysed using one-way analysis of variance (ANOVA) of different sorghum varieties and fermentation times (*P* < 0.05). The difference between the mean values was determined using the least significant difference (*P* < 0.05), which was indicated by different letters. Origin 2018 was used to draw the figures, including those for physicochemical properties and microbial communities.

## Results and Discussion

### Aroma Compound of Liquor

**FIGURE 1:**
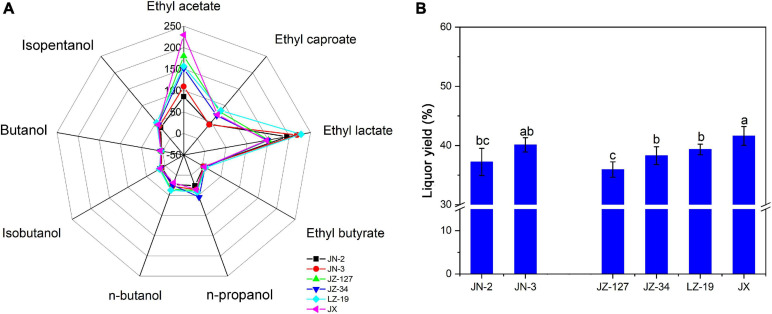
Volatile compounds of liquor **(A)** and liquor yield **(B)** in two waxy (JN-2 and JN-3) and four non-waxy sorghum varieties (JZ-127, JZ-34, LZ-19, and JX). Different letters indicate significant differences (ANOVA, *P* < 0.05) amongst various sorghum varieties.

On the basis of aroma characteristics, Chinese liquor can be classified into strong aroma type, light aroma type, soy sauce aroma type, sweet honey type, and miscellaneous types (Fan and Qian, 2006). Liquor aroma compounds exist in various proportions to form different flavours and types of liquor, thus improving the quality of liquor production. Ester is the most abundant and important aromatic component in liquor; the content of esters determines the flavour characteristics of liquor (Lan et al., 2017). To identify and quantify the aroma compounds responsible for the overall aroma profile of liquor, Xifeng liquor was analysed by LLME-GC-MS. Nine aroma compounds including ethyl acetate, ethyl caproate, ethyl lactate, ethyl butyrate, n-propanol, n-butanol, isobutanol, butanol, and isoamylol (Figure 1A) were identified and quantitated in Xifeng liquor. The contents of ethyl acetate, ethyl caproate, ethyl lactate and ethyl butyrate ranged from 85.86 to 229.29 mg/100 mL, 42.18 to 84.66 mg/100 mL, 146.00 to 227.98 mg/100 mL, and 3.27 to 7.44 mg/100 mL, respectively. Obviously, ethyl acetate had the highest content (229.29 mg/100 mL) in JX, which was commonly regarded as the characteristic aroma compound of Xifeng liquor (Cao et al., 2017). Ethyl esters with a fruity aroma are often considered as the major contributors to the aroma profile of Chinese Baijiu (Li et al., 2021). Apart from ethyl acetate, ethyl lactate accounted for half of the proportion of ethyl acetate in JX (Figure 1A), indicating that Xifeng liquor has the characteristic miscellaneous type of mixed flavour liquor and aroma with ethyl acetate as the main aroma compound (Hu et al., 2011; Fan et al., 2019). In addition, JN-2, JN-3, JZ-127, JZ-34, and LZ-19 contained significantly higher amounts of ethyl lactate than JX. A moderate ethyl lactate content is beneficial for the liquor type; however, excessive amounts can cause astringent liquor taste and deteriorated quality (Liu et al., 2011). Although the abundance of amylopectin in waxy sorghum promotes swelling and gelatinisation, it is suitable for liquors with strong aroma and soy sauce aroma types. Therefore, JX as a non-waxy sorghum is suitable for use in the production of miscellaneous type Xifeng liquor in Shaanxi. These results are similar to those of previous studies (Nambou et al., 2014; Corona et al., 2016). Esters are found in high amounts after fermentation, moreover, some major esters have strong relations with fruity/floral/green aromas and are mainly produced by yeasts.

Given that the inherent esters in Chinese liquor are mainly produced during the fermentation stages through the esterification of alcohols and acids, alcohols have a significant influence on the yield and intensity of aroma esters (González-Rompinelli et al., 2013; Wang et al., 2015). In the present study, the contents of n-propanol, n-butanol, isobutanol, butanol and isopentanol ranged from 26.06 to 54.53 mg/100 mL, 18.73 to 37.25 mg/100 mL, 9.31 to 15.23 mg/100 mL, 2.40 to 4.90 mg/100 mL, and 33.42 to 48.37 mg/100 mL, respectively, which are lower than most of the ester compounds (Figure 1A), due to their low odour threshold values. Similar to the contents of ester, suitable contents of the five alcohol compounds, especially n-propanol, were detected in JN-3 and JX. Notably, JX had higher contents of isoamylol and isobutanol than JN-3. Given that alcohols have typical fruity and floral aromas, they are used not only as alcoholic sweeteners and flavouring agents but also as precursors of flavouring substances, which have certain effects on liquor flavour (Lan et al., 2017). For example, as expected with any alcoholic beverage, alcohols are amongst the major volatile compounds. During fermentation, yeast can form alcohols from sugars under aerobic conditions and from amino acids under anaerobic conditions (Wondra and Berovic, 2001). However, the alcohol content needs to be controlled within a certain range because slight changes in it will remarkably affect the type of traditional liquor, resulting in spicy, bitter and astringent flavours. Compared with other Chinese Baijiu, the contents of the nine aroma compounds mentioned above in the test Xifeng liquor were different from those of Gujinggong, Jiannanchun, Luzhou Laojiao, Wuliangye, and Yanghe Daqu (Xiao et al., 2014; Zheng et al., 2014a). This discrepancy in the contents of aroma compounds leads to the delicate difference of smell and taste of Xifeng liquor from other Chinese Baijiu. JX and JN-3 exhibited a higher liquor yield than the other sorghums. However, the highest liquor yield was observed in JX (Figure 1B).

### Physicochemical Parameters in Fermented Grains

**FIGURE 2:**
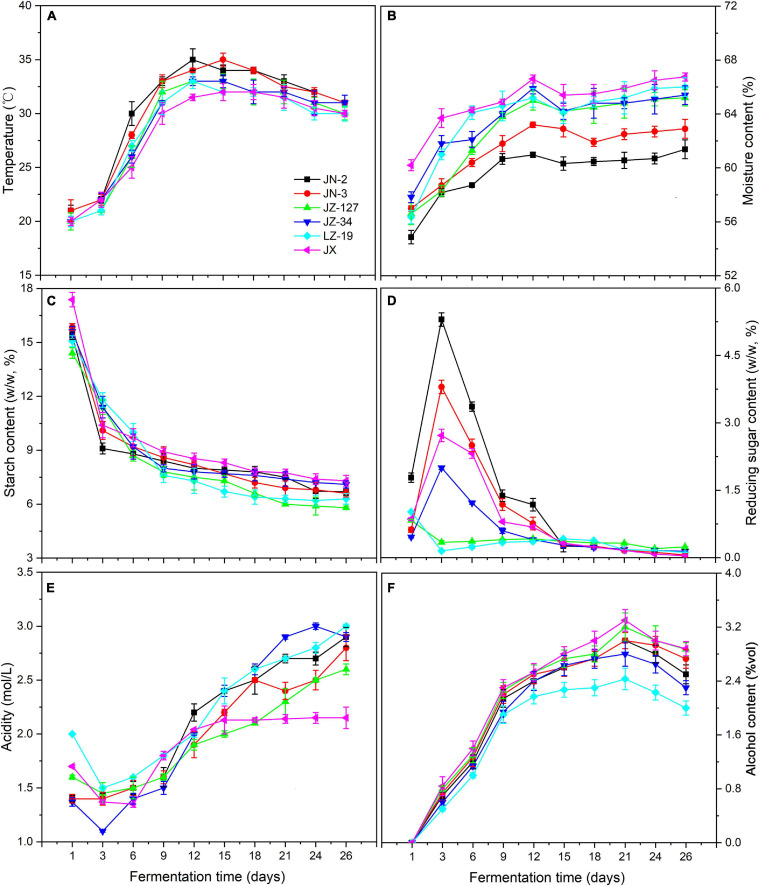
Changes in temperature **(A)**, moisture content **(B)**, starch content **(C)**, reducing sugar content **(D)**, acidity **(E)**, and alcohol content **(F)** of fermented grains during fermentation in two waxy (JN-2 and JN-3) and four non-waxy sorghum varieties (JZ-127, JZ-34, LZ-19, and JX).

Chinese liquor is a traditional distillate fermented from grains. Not only are fermented grains the main body of brewing liquor, but also the microorganisms in fermented grains drive the formation of flavour substances (Jin et al., 2017). Elucidating the dynamic changes of parameters in fermented grains is important for liquor production. The starch in fermented grains can be degraded and converted into reducing sugars, alcohols, acids, esters, aldehydes, and ketones, which affect the quality and type of liquor. Meanwhile, the transmission of environmental information is crucial for the regulation of changes in microbial species, formation and accumulation of metabolites, and determination of the direction of material and energy metabolism. Therefore, after fermentation, the fresh liquor is distilled out and then aged under controlled conditions. The aged distillate is adjusted to the designated alcohol content and blended to ensure the quality of the finished product (Shen, 1996). The physicochemical parameters of fermented grains obtained in this study, including temperature, moisture, starch, reducing sugar, acidity, and alcohol (Figure 2 and Supplementary Table 2), are presented and discussed in the following.

The micro-environment in pits changes during the fermentation process, coupled with the utilisation of nutrients and accumulation of metabolic products, such as decrease in pH and increase of alcohol concentration. In addition, environmental factors, such as temperature and moisture, affect the performance of traditional liquor fermentation. It is crucial to optimise the environmental conditions for co-culture fermentation. Not only are these factors vital indicators of microorganism growth and metabolism; they also affect the bioactivity of microorganisms (Wu et al., 2013).

In the present study, temperature and moisture constantly increased, but the former decreased after 12 days of fermentation. The temperatures of waxy sorghums (JN-2 and JN-3) were higher than those of non-waxy sorghums (JZ-127, JZ-34, LZ-19, and JX) (Figure 2A), thereby accelerating the oxygen utilisation, propagation and death of microorganisms (Wang and Xu, 2015; Yan et al., 2015). On the other hand, higher temperature also leads to greater loss of esters due to the increased rates of hydrolysis and volatilisation, whilst a lower fermentation temperature favours the formation of short-chain esters (Fan and Qian, 2006). Other environmental factors such as moisture are not only indispensable to the biochemical reactions in the fermentation process, but also ensure the growth of microbes and provide an effective solvent for their metabolites such as alcohols, acids and esters. Moisture also affects the output rate of the liquor and the extraction of aroma substances existing in fermented grains during the fermentation process. Compared with the temperature, the moisture content was approximately 60% (*P <* 0.05). The non-waxy sorghums (JZ-127, JZ-34, LZ-19, and JX) exhibited higher moisture contents than their waxy counterparts (JN-2 and JN-3) (Figure 2B). Meanwhile, the highest moisture content was maintained in JX fermented grains.

During fermentation, starch as substrate is fermented to sugar and then the sugar forms different compounds (Olofsson, 2008). Starch saccharification rates also produce different sugar concentrations. When starch is less inhibited by reducing sugars in enzymatic hydrolysis, the efficiency of substrate utilisation is increased, thereby significantly influencing cell growth and the metabolism of flavour compounds. The reducing sugar content represents the balance between starch saccharification and sugar consumption. The sugar consumption rate is regulated by the saccharification rate. These factors ultimately affect the final alcohol yield and quality of the liquor (Wu et al., 2015). In the present study, the starch content of fermented grains exhibited an overall downward trend in all sorghum varieties, and the changes in reducing sugars in waxy (JN-2 and JN-3) and non-waxy (JZ-127, JZ-34, LZ-19, and JX) sorghums were notably different. Moreover, JX sorghum contained the highest starch content amongst the sorghum varieties during fermentation (Figures 2C,D). High starch content is beneficial for microbial growth. Starch can be degraded and converted into reducing sugars and other substances. We found that the content of reducing sugars increased 3 days before fermentation and reached a peak, except for that in JZ-127 and LZ-19. This phenomenon was due to the rich oxygen and nutrients in the early stage of fermentation, and microorganisms grew and reproduced rapidly, which resulted in the production of high amounts of amylases and saccharification enzymes and increase in the reducing sugar content (Mcfeeters, 2004). Furthermore, reducing sugars are metabolised by yeast to produce alcohol, thereby affecting the quality and type of liquors (Olofsson, 2008; Chen et al., 2014).

During water kefir fermentation, low nutrient concentrations cause a slow fermentation, resulting in high total residual carbohydrate concentrations and high pH values (Laureys et al., 2018). In liquor production, the environmental factor pH affects the performance of traditional liquor fermentation. Not only is pH an important indicator of microorganism growth and metabolism; it also affects the bioactivity of microorganisms (Wu et al., 2013). In this study, the acidity of fermented grains decreased substantially 3 days before fermentation (Figure 2E). The amplification of acidity reached the maximum on the 9th to 12th day of fermentation. The acidity values in JN-3, JN-2, JZ-127, JZ-34, LZ-19, and JX sorghum increased by 37.5, 18.8, 18.8, 33.3, 13.3, and 11.1%, respectively. The acidity of fermented grains showed an upward trend from day 12 to day 26 of fermentation. With the consumption of nutrients such as oxygen, acid-producing bacterial metabolism produced a certain acidity during this stage. A suitable acidity is not only conducive to starch gelatinisation and saccharification but also inhibits the growth of bacteria and facilitates the growth and reproduction of yeast (Wang and Xu, 2015; Yan et al., 2015). Distinctively, we found that JX fermented grains always had low acidity, which is consistent with a previous report (Wang et al., 2014). Wang et al. (2014) demonstrated that in the brewing processes of Chinese liquors, the extreme environment made the dominant bacteria prefer conditions with high ethanol concentrations and low pH values.

Alcohol is the main product of Chinese liquor fermentation, and its concentration is one of the key factors reflecting the fermentation state. During fermentation, yeast can form alcohols from sugars and amino acids under aerobic and anaerobic conditions, respectively (Wondra and Berovic, 2001). Despite the low ethanol content in the fermenting liquid of *Prunus mahaleb* fruit, it contributed to enhancing the sensory characteristics of fermented products and had a preservative function as it inhibited the development of unwanted microorganisms (Swiegers et al., 2005; Gerardi et al., 2019). In this study, the alcohol content showed an upward trend on days 1–21 of fermentation, and alcohol was produced rapidly from day 1 to day 9 (Figure 2F). Moreover, the alcohol contents of non-waxy sorghums (JZ-127, JZ-34, LZ-19, and JX) were higher than those of waxy varieties (JN-2 and JN-3); the highest content was observed in JX, indicating that additional substrates for esterification reactions were produced and/or the utilisation of fatty acids was related to the production of alcohol by several microbes (Gong et al., 2017). In the late fermentation stage, the alcohol content showed a decreasing trend, which was possibly due to the inhibited yeast growth under the poor environment following the acid-producing period; subsequently, a portion of the alcohol was converted into acids and esters (Richter et al., 2013). The supply of monosaccharides as a carbon source material for consumption by microorganisms is insufficient.

### Microbial Community Diversity in Fermented Grains

Chinese liquor is produced from grains with mixed microbial fermentation technique in solid form, and the metabolic products of the microorganism are important for liquor quality. The micro-environment changes will alter the quantity and species of microorganisms in fermented grains. Therefore, the microorganism community structure of fermented grains not only incarnates the micro-environment in pits, but also affects the formation of liquor flavour components. Studies have shown that the structural diversity and changes of microbial communities are involved in the fermentation of Chinese liquor by multiple analytical methods (Wang X. et al., 2017). To understand the microbial community changes during fermentation, the microbial communities in fermented grains collected from different days were revealed using 16S and ITS Illumina MiSeq technique. After quality sequencing, bacterial (1,623,526 sequences) and fungal communities (761,024 paired-end sequences) were obtained using the 338F/806R (bacterial 16S rRNA) and ITS1F/ITS2 (fungal ITS) primer sets across all fermented grain samples. The numbers of bacterial and fungal sequences varied from 110,328 to 192,689 (mean = 135,586) and from 43,715 to 83,579 (mean = 63,653) per sample, respectively. For downstream analyses of bacterial and fungal sequences, the datasets were rarefied to 108,918 and 41,548 sequences, respectively. From these data, we could conclude that the numbers in bacterial communities were higher than those in fungi.

**FIGURE 3:**
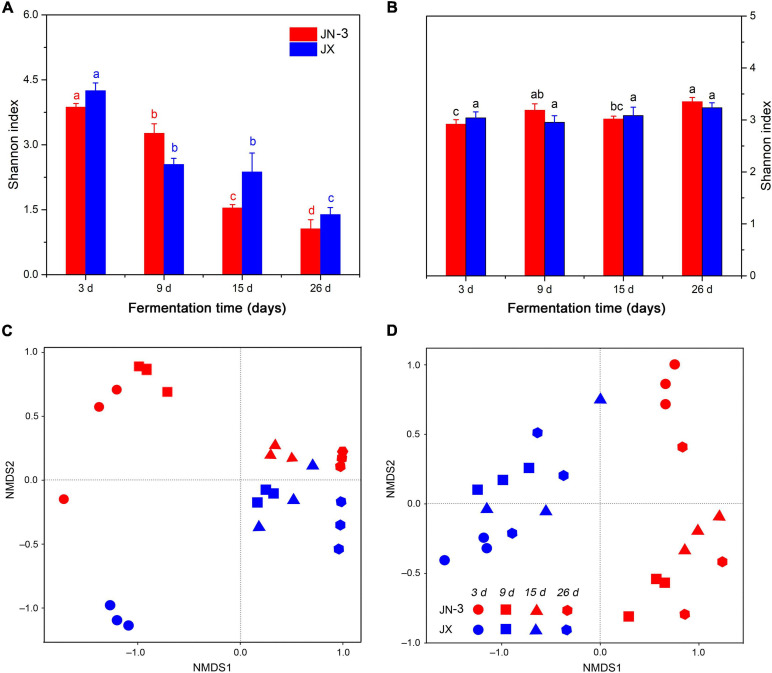
Changes in microbial alpha diversity (Shannon index) [**(A)**, bacteria; **(B)**, fungi] and beta diversity (NMDS) [**(C)**, bacteria; **(D)**, fungi] of fermented grains in one waxy (JN-3) and one non-waxy sorghum (JX). Different letters indicate significant differences (ANOVA, *P* < 0.05) amongst different fermentation times for the same sorghum variety.

An OTU level approach was used to calculate the microbial diversity in different sorghum varieties. The Shannon index, which represents the abundance and diversity of microbial communities, was used to reflect the microbial alpha diversity (Gong et al., 2019). As shown in Figure 3A, the bacterial alpha diversity had a more remarkable difference in JX than in JN-3, indicating the rich bacterial diversity of the former. Moreover, the microbial diversity (Shannon) indices of the microbial communities in fermented grains decreased significantly from day 3 to day 26. At the beginning of fermentation, a high diversity of microorganisms was observed in fermented grains. With the fermentation time, many microorganisms could not endure the changes of the micro-environment in pits, leading to their death. The fungal alpha diversity showed negligible differences in JN-3 and JX after fermentation (Figure 3B). NMDS was used to reflect the microbial beta diversity amongst the sorghum varieties (Figures 3C,D). The largest difference in bacterial beta diversity was observed in JN-3 and JX after 3 days of fermentation, revealing that most of the changes occurred in the bacterial community between these varieties. The same trend was not observed for fungi. Our results confirmed that the bacterial alpha diversity was higher in fermented grains of JX, but the fungal alpha diversity for JN-3 and JX fermented grains was difficult to predict, indicating that the bacterial community had higher sensitivity to the Xifeng liquor system than the fungal community.

### Bacterial Community Composition in Fermented Grains

As important components of microorganisms for brewing liquor, bacteria can be applied to regulate liquor production and improve liquor quality (Wang X. et al., 2017). In this study, bacterial microorganisms at the phyla level mainly included Firmicutes, Proteobacteria, Bacteroidetes, and Actinobacteria (Table 1). Previous research showed that the environment of the fermentation pit was rich in bacteria mainly belonging to Firmicutes, Actinobacteria and Proteobacteria (Hu et al., 2016). The results of the present study were therefore consistent with those of previous reports. Firmicutes accounted for the total proportion of bacteria in JN-3 and JX, with a consistently increasing trend from day 3 to day 26, whereas JX had a significantly higher bacterial content (46.75 and 15.10%) than JN-3 during the early stage of fermentation (on the 3rd and 9th day of fermentation, respectively). Proteobacteria, Bacteroidetes, and Actinobacteria showed opposite trends during fermentation, and their total proportion in JN-3 and JX consistently declined. Bacilli was the main class observed in all sorghum varieties (Table 1). This class has a strong ability to secrete protease, amylase and cellulase and decomposes macromolecular substances to form flavour compounds, such as nitrogen-containing substances. Bacilli also shows high temperature resistance, enzyme production and fragrance production during accumulation (Zhao et al., 2017). In a previous study, the bacterial communities in the fermented grains of two Chinese liquor types were compared (Wang H. Y. et al., 2008), and classes such as Bacilli, Bacteroidetes and Clostridia were dominant in strong flavour type fermented grain. Zheng et al. (2014b) found Lactobacillales as the dominant order in fermented grains, which is consistent with our results. Lactobacillales dominated the fermentation process with relative abundances of 18.35–96.74 and 49.03–98.15% in JN-3 and JX, respectively. Moreover, the proportion of Lactobacillales in JX was higher than that in JN-3 during the whole process of fermentation (Table 1). The decreasing trend of prokaryotic diversity and the predominance of *Lactobacillus* may be attributed to the rapid production of Lactobacillales and their tolerance to high concentrations of lactic acid and ethanol in the fermentation process (Li et al., 2011; Martınez-Torres et al., 2016). The inhibition of prokaryotes without acid- and alcohol-resistant properties may also protect the liquor fermentation process from microbiological contamination (Wang X. et al., 2017). Meanwhile, the changes in these bacteria are consistent with those in starch and reducing sugar contents, thus changing the microbial activity to improve the use of nutrients in fermented grains (Sundberg et al., 2013).

**TABLE 1:** Relative abundances (average values and standard error) of bacterial compositions across taxonomical classification (phyla, class, and order) of fermented grains in waxy and non-waxy sorghum during fermentation.

**Phyla**	**Class**	**Order**	**3 days**		**9 days**		**15 days**		**26 days**	
	
			**JN-3**	**JX**	**JN-3**	**JX**	**JN-3**	**JX**	**JN-3**	**JX**
Firm			42.96 ± 1.85d	63.04 ± 3.48d	68.63 ± 0.95c	78.99 ± 0.38c	91.40 ± 0.34b	91.65 ± 1.32b	98.09 ± 0.26a	99.18 ± 0.15a
	Bacil		19.77 ± 0.55d	51.22 ± 1.76d	27.28 ± 0.58c	70.04 ± 0.70c	77.55 ± 0.49b	84.61 ± 1.30b	97.28 ± 0.55a	98.39 ± 0.30a
		Lactob	18.35 ± 0.96d	49.03 ± 2.73d	26.61 ± 0.52c	69.36 ± 0.61c	77.49 ± 0.49b	83.95 ± 1.81b	96.74 ± 0.85a	98.15 ± 0.60a
		Bacill	1.40 ± 0.94a	2.18 ± 1.01a	0.67 ± 0.07ab	0.51 ± 0.11b	0.06 ± 0.02b	0.13 ± 0.05b	0.07 ± 0.04b	0.08 ± 0.02b
	Clost		22.11 ± 1.79b	10.51 ± 1.77a	39.47 ± 1.34a	7.99 ± 0.72b	12.84 ± 0.64c	6.27 ± 0.25b	0.48 ± 0.02d	0.50 ± 0.03c
		Clostr	22.11 ± 1.79b	10.51 ± 1.77a	39.47 ± 1.34a	7.99 ± 0.72b	12.84 ± 0.64c	6.27 ± 0.25b	0.48 ± 0.02d	0.50 ± 0.03c
	Erysi		0.55 ± 0.33b	1.06 ± 0.05a	1.01 ± 0.27a	0.67 ± 0.20b	0.14 ± 0.04b	0.37 ± 0.20c	0.14 ± 0.01b	0.04 ± 0.01c
		Erysip	0.55 ± 0.33b	1.06 ± 0.05a	1.01 ± 0.27a	0.67 ± 0.20b	0.14 ± 0.04b	0.37 ± 0.20c	0.14 ± 0.01b	0.04 ± 0.01c
Prot			23.51 ± 1.48a	21.78 ± 4.08a	5.95 ± 1.05b	11.50 ± 0.17b	1.17 ± 0.06c	2.88 ± 0.94c	1.08 ± 0.02c	0.41 ± 0.03c
	Alpha		21.95 ± 1.13a	20.54 ± 4.07a	3.27 ± 1.05b	11.12 ± 0.14b	0.70 ± 0.04c	2.48 ± 0.93c	0.62 ± 0.02c	0.22 ± 0.01c
		Rhodos	21.23 ± 0.79a	19.94 ± 3.97a	2.27 ± 0.72b	10.97 ± 0.13b	0.42 ± 0.09c	2.09 ± 1.01c	0.24 ± 0.02c	0.17 ± 0.04c
	Gamma		1.19 ± 0.15b	1.08 ± 0.03a	1.88 ± 0.35a	0.34 ± 0.03b	0.42 ± 0.09c	0.35 ± 0.04b	0.36 ± 0.05c	0.17 ± 0.02c
Bact			29.18 ± 1.12a	9.85 ± 1.26a	21.57 ± 1.37b	5.35 ± 0.50b	6.76 ± 0.20c	2.96 ± 0.48c	0.34 ± 0.08d	0.18 ± 0.03d
	Bacte		28.42 ± 1.56a	9.67 ± 1.29a	20.99 ± 1.54b	5.30 ± 0.50b	5.25 ± 0.93c	2.83 ± 0.43c	0.15 ± 0.03d	0.16 ± 0.02d
		Bacter	28.42 ± 1.56a	9.67 ± 1.29a	20.99 ± 1.54b	5.30 ± 0.50b	5.25 ± 0.93c	2.83 ± 0.43c	0.15 ± 0.03d	0.16 ± 0.02d
Acti			4.09 ± 0.56a	4.73 ± 0.60a	3.66 ± 0.33a	3.95 ± 0.18b	0.56 ± 0.03b	2.11 ± 0.48c	0.25 ± 0.03b	0.22 ± 0.09d
	Actin		4.03 ± 0.59a	4.64 ± 0.60a	3.57 ± 0.35a	3.93 ± 0.18a	0.53 ± 0.03b	2.08 ± 0.46b	0.24 ± 0.03b	0.22 ± 0.09c

**Phyla level*: Firmicutes (Firm), Proteobacteria (Prot), Bacteroidetes (Bact), and Actinobacteria (Acti). *Class level*: Bacilli (Bacil), Clostridia (Clost), Erysipelotrichia (Erysi), Alphaproteobacteria (Alpha), Gammaproteobacteria (Gamma), Bacteroidia (Bacte), and Actinobacteria (Actin). *Order level*: Bacillales (Bacill), Lactobacillales (Lactob), Clostridiales (Clostr), Erysipelotrichales (Erysip), Rhodospirillales (Rhodos), and Bacteroidales (Bacter). Different letters indicate significant differences (ANOVA, *P* < 0.05) among different fermentation time for the same sorghum variety.*

**FIGURE 4:**
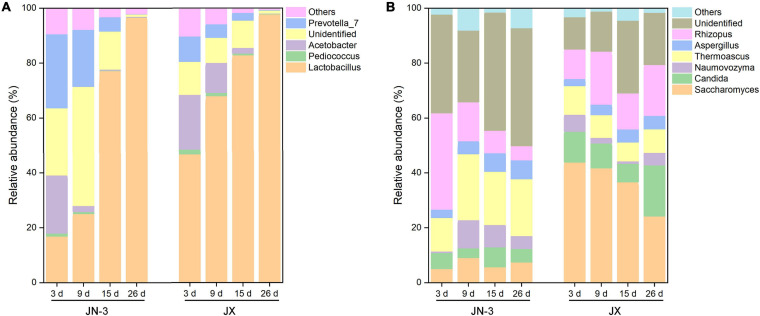
Changes in bacterial **(A)** and fungal **(B)** taxonomic composition of fermented grains at the genus level in one waxy (JN-3) and one non-waxy sorghum (JX). The abundance of each taxon was calculated as the percentage of sequences per gradient for a given microbial group. The taxa represented the class that occurred over 1% abundance in at least one type of sample.

At the genus level, four main bacteria, including *Lactobacillus*, *Pediococcus*, *Acetobacter*, and *Prevotella_7*, were detected (Figure 4A). In particular, the proportion of *Lactobacillus* gradually increased during fermentation. Our results were consistent with those of a previous study on the bacterial community structures and changes in fermented grain (Li et al., 2011; Wang X. et al., 2017). There into, *Acetobacter* is a strict aerobe, but *Lactobacillus* and *Pediococcus* are facultative aerobes. When fermented for 9 days, the abundance of *Lactobacillus* increased to 77.96% in JX, but it only increased to 23.45% in JN-3. Meanwhile, the abundance of other genera decreased in JX and JN-3 during fermentation, but it was higher in JX than in JN-3. At the end of fermentation, only *Lactobacillus* was abundant in fermented grain. In our study, the highest ethyl acetate content (229.29 mg/100 mL) and highest liquor yield were found in JX. Their findings resulted from bacterial community changes. *Lactobacillus* is the most important bacterium contributing to the fermentation process of producing traditional foods and beverages (Liu et al., 2011). *Acetobacter* is the main functional bacteria used for producing vinegar (Wu et al., 2012). This genus is more competitive under acidic conditions (Jung et al., 2012; Li et al., 2016). In the middle and late stages of fermentation, *Lactobacillus* occupied the majority of microorganisms in the fermented grains, which promoted acidity to rapidly increase the level of esterase and effectively inhibit the metabolic activities of other bacteria (Zheng et al., 2014). *Lactobacillus* can also use lactic acid to form aroma precursors, such as acetic acid, propionic acid and butyric acid, resulting in the synthesis of various esters that increase the aroma components in liquor (Li et al., 2011). However, excessive amounts of *Lactobacillus* in the liquor will result in excessive lactic acid levels and ethyl lactate, resulting in poor taste (Liu et al., 2011). Therefore, the quantity and activity of *Lactobacillus* should be controlled during fermentation to ensure the liquor quality.

### Fungal Community Composition in Fermented Grains

Xifeng liquor is produced through a typical simultaneous saccharification and fermentation process. During simultaneous saccharification and fermentation, starch is hydrolysed to fermentable sugar by glucoamylase and α-amylase, which could be produced by many fungal species, such as *Aspergillus*, *Paecilomyces*, *Rhizopus*, *Monascus*, and *Penicillium*. In the meantime, these filamentous fungi have been reported to serve as saccharifying agents (Nahar et al., 2008; Lv et al., 2012; Chen et al., 2014). Table 2 shows a relatively simple distribution of the taxonomic structure observed at the phyla level. The dominant fungi at the phyla level were Ascomycota and Mucoromycota, accounting for nearly the total proportion of fungi. Moreover, Ascomycota accounted for the total proportion of fungi in JX, which was significantly higher than that in JN-3 during fermentation. Ascomycota was the most dominant fungi at the phyla level, providing the driving force for liquor fermentation. The proportion of Ascomycota consistently increased, whereas that of Mucoromycota gradually decreased. Mucoromycota has strong protease activity, thus allowing the digestion of proteins in dregs into amino acids and further reaction with reducing sugars to form various aroma substances. Saccharomycetes and Saccharomycetales were the main fungal class and order, respectively. Saccharomycetes members play important roles in the synthesis of caproic acid and butyric acid, implying that they also play key roles in brewing liquor (Minnaar et al., 2017).

**TABLE 2:** Relative abundances (average values and standard error) of fungal compositions across taxonomical classification (phyla, class, and order) of fermented grains in waxy and non-waxy sorghum during fermentation.

**Phyla**	**Class**	**Order**	**3 days**		**9 days**		**15 days**		**26 days**	
	
			**JN-3**	**JX**	**JN-3**	**JX**	**JN-3**	**JX**	**JN-3**	**JX**
Asco			64.29 ± 1.41d	89.06 ± 0.67a	85.33 ± 0.60c	79.85 ± 1.43b	91.08 ± 1.15b	86.34 ± 1.47a	94.63 ± 0.22a	81.11 ± 2.28b
	Sacch		48.51 ± 3.17c	75.66 ± 0.43a	56.01 ± 3.45b	61.19 ± 4.35c	63.86 ± 1.83a	74.09 ± 1.18a	65.46 ± 2.12a	67.23 ± 3.25b
		Saccha	48.51 ± 3.17c	75.66 ± 0.43a	56.01 ± 3.45b	61.19 ± 4.35c	63.86 ± 1.83a	74.09 ± 1.18a	65.46 ± 2.12a	67.23 ± 3.25b
	Eurot		15.36 ± 1.77b	13.35 ± 0.25b	28.80 ± 3.56a	18.54 ± 0.81a	26.39 ± 2.31a	11.61 ± 0.45c	27.97 ± 1.71a	13.77 ± 1.03b
		Euroti	15.36 ± 1.77b	13.35 ± 0.25b	28.80 ± 3.56a	18.54 ± 0.81a	26.39 ± 2.31a	11.61 ± 0.45c	27.97 ± 1.72a	13.77 ± 1.03b
Muco			35.59 ± 1.43a	10.81 ± 0.60b	14.26 ± 0.74b	19.96 ± 1.61a	8.26 ± 0.97c	13.39 ± 1.50b	5.18 ± 0.09d	18.73 ± 2.23a
	Mucor		35.59 ± 1.43a	10.81 ± 0.60b	14.26 ± 0.74b	19.62 ± 1.61a	8.26 ± 0.97c	13.39 ± 1.50b	5.18 ± 0.09d	18.73 ± 2.23a
		Mucora	35.59 ± 1.43a	10.81 ± 0.60b	14.26 ± 0.74b	19.62 ± 1.61a	8.26 ± 0.97c	13.39 ± 1.90b	5.18 ± 0.09d	18.73 ± 2.23a

**Phyla level*: Ascomycota (Asco) and Mucoromycota (Muco). *Class level*: Saccharomycetes (Sacch), Eurotiomycetes (Eurot), and Mucoromycetes (Mucor). *Order level*: Saccharomycetales (Saccha), Eurotiales (Euroti), and Mucorales (Mucora). Different letters indicate significant differences (ANOVA, *P* < 0.05) among different fermentation time for the same sorghum variety.*

The changes in fungal communities at the genus level are shown in Figure 4B. Six primary fungal genera, including *Saccharomyces*, *Candida*, *Rhizopus*, *Thermoascus*, *Naumovozyma*, and *Aspergillus*, were observed. The starch in sorghum cannot be directly utilised by most yeasts and bacteria and needs to be hydrolysed into fermentable reducing sugars by α-amylase, β-amylase, glucoamylase and protease. Importantly, these enzymes are mainly produced by a series of microorganisms, such as *Aspergillus*, *Rhizopus*, *Bacillus* spp., *Lactobacillus*, *Wickerhamomyces*, and *Saccharomycopsis* (Beaumont, 2002; Zheng et al., 2011; Chen et al., 2014). Therefore, these genera can effectively decompose starch and increase the content of low-molecular sugars during fermentation to promote liquor fermentation. We found that *Saccharomyces* accounted for one half of the total fungal proportion at the genus level, with the proportion in JX being significantly higher than that in JN-3 during fermentation. *Saccharomyces* is a dominant fungus and has served as a fermenting agent to convert fermentable substrates into alcohol. However, the proportion of *Saccharomyces* in JN-3 was extremely low during the whole fermentation process, which suggests that JN-3 may be unsuitable for brewing liquor. Although several low-abundance genera, such as *Aspergillus* and *Naumovozyma*, showed no significant difference in JX and JN-3, they also played a vital role in the liquor flavour-making process. Wang H. Y. et al. (2008) observed that *Aspergillus* spp. contributed to the saccharification of starch, which accords with the significantly negative relationship between the starch content and *Aspergillus* in this study (Supplementary Table 4). Moreover, Vivier et al. (1997) stated that 150 yeast species, expressing glucoamylase in addition to α-amylase with debranching activity, can degrade starch with great efficacy for carbon and energy sources. Thus, fungal communities (*Saccharomyces*, *Candida*, *Rhizopus*, *Thermoascus*, *Naumovozyma*, and *Aspergillus*) are considered as functional microbiota that produce a range of lytic enzymes for synthesising substrates for liquor fermentation and further formation of flavour compounds. Similar results were also reported for *Saccharomyces* and *Candida* in glucose-supplemented minimal medium to understand the role of carbon sources in developing table grape sour rot (Gerardi et al., 2019; Pinto et al., 2019).

### Correlation Between Physicochemical Parameters and Microbial Compositions

**FIGURE 5:**
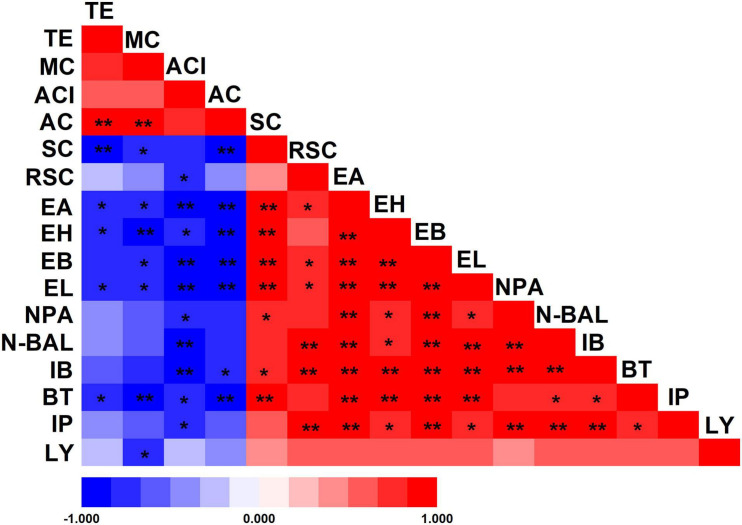
Pearson’s correlation coefficients between physicochemical properties of fermented grains and volatile compounds of liquor. *Correlation is significant at the 0.05 level. **Correlation is significant at the 0.01 level. TE, temperature; MC, moisture content; ACI, acidity; AC, alcohol content; SC, starch content; RSC, reducing sugar content; EA, ethyl acetate; EH, ethyl hexanoate; EB, ethyl butyrate; EL, ethyl lactate; NPA, n-propanol; N-BAL, n-butanol; IB, isobutanol; BT, butanol; IP, isopentanol; LY, liquor yield.

**FIGURE 6:**
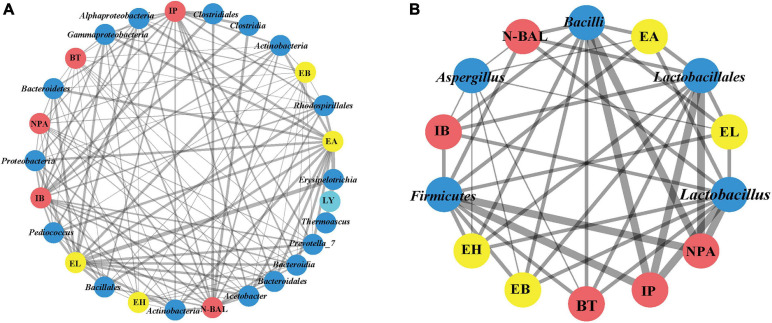
Co-occurrence network of bacterial and fungal communities (phyla, class, order, and genus) of fermented grains and volatile compounds of liquor based on correlation analysis. A connection stands for a strong positive [**(A)**, Spearman’s ρ > 0.6] or negative [**(B)**, Spearman’s ρ > –0.6] correlation. The thickness of the line is proportional to the absolute value of Spearman’s correlation coefficients.

The differences in the environment also play a key role in the selection of microorganisms used for fermentation. As a result of these multiple variations, Wuliangye, Jiannanchun, and Yanghe Daqu all have unique aroma profiles (Shen, 1996; Fan and Xu, 2000). Therefore, we investigated the interactions between liquor quality and physicochemical property and microbial compositions during fermentation by using Pearson’s correlation coefficients (Figures 5, 6). The results exhibited that temperature, moisture and acidity were negatively related to alcohol and ester, whereas starch and reducing sugars showed a positive relation.

These liquor fermentation parameters were also related to the relative abundances of bacterial and fungal communities (Supplementary Tables 2,3). Amongst these parameters, temperature, moisture, acidity and alcohol content were negatively related to the relative abundances of bacterial communities, except for Firmicutes, Bacilli and Lactobacillales, in contrast with the relation to starch and reducing sugars. Hence, large amounts of starch and reducing sugar were consumed by Lactobacillales. Temperature, moisture and alcohol content were positively related to the relative abundances of fungal communities, except for Mucoromycota, Mucoromycetes, and Mucorales, in contrast with the relation to starch and reducing sugar.

We found that Proteobacteria, Bacteroidetes and Actinobacteria; Alphaproteobacteria, Bacteroidia, and Actinobacteria; Bacillales, Bacteroidales, and Rhodospirillales; and *Acetobacter*, *Pediococcus*, and *Prevotella_7* were positively related to four esters and five alcohols [the absolute value of Spearman’s rank correlation coefficient (ρ) was over 0.6] (Figure 6). Despite the low relative abundances of *Acetobacter*, Actinobacteria, Bacillales, and *Pediococcus* in bacterial communities, they played important roles during fermentation. Meanwhile, alcohol and ester were significantly negatively related to Firmicutes, Bacilli, Lactobacillales, and *Lactobacillus* [the absolute value of Spearman’s rank correlation coefficient (ρ) was over 0.6]. As lactic acid bacteria, *Lactobacillus*, *Leuconostoc*, *Lactococcus*, *Pediococcus*, and *Weissella* are regarded as the main functional genera in the fermentation process of Chinese liquor (Zhang et al., 2005; Li et al., 2011). Previous studies showed that lactic acid bacteria can produce lactic acid, and *Gluconobacter* and *Acetobacter* can produce acetic acid (Jung et al., 2012; Li et al., 2016). High concentrations of lactic acid and acetic acid in fermented grains resulted in acidic stress. Meanwhile, the decreased diversity during fermentation also confirmed the selective action on microbes in fermented grains. Lactic acid is a well-known precursor of ethyl lactate, which can enhance the mellow feeling of Chinese liquor. By contrast, acetic acid is the precursor of ethyl acetate, which contributes to the fruit flavour of Chinese liquor (Gao et al., 2014). These data demonstrate the relationship between alcohol esters and microorganisms.

For fungal communities, *Saccharomyces* and *Rhizopus* were positively related to four esters and five alcohols, except for Mucoromycota, Mucoromycetes, and Mucorales. However, no significant differences were observed (Supplementary Table 5). Notably, only *Aspergillus* was significantly negatively related to four esters (EA, EH, EB, and EL) and four alcohols (NPA, N-BAL, IB, and BT) [the absolute value of Spearman’s rank correlation coefficient (ρ) was over 0.6].

**FIGURE 7:**
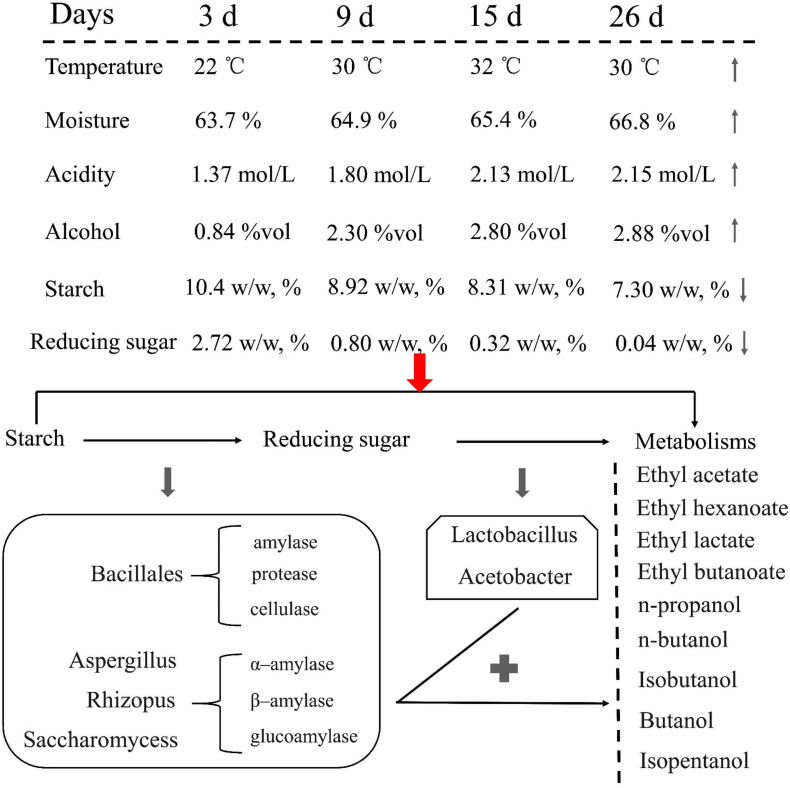
Changes in the physicochemical parameters of JX fermented grains and hypothetical flux of carbon and involvement of key microorganisms during liquor fermentation from starch to the main metabolic products.

Ester and alcohol have a direct relationship with the fermentation environment and microbial communities and are involved in the fermentation process. In particular, the changes in physical and chemical properties, such as the use of starch and reducing sugars, should be considered during fermentation. Comprehensive analysis showed that JX sorghum is suitable for Xifeng liquor production. Subsequently, the production of Xifeng liquor under the fermentation conditions of JX sorghum as the standard can be promoted (Figure 7).

## Conclusion

This study investigated the dynamic changes in the physicochemical parameters of JX fermented grains. Although the fungal beta diversity remained almost unchanged during fermentation, higher bacterial alpha diversity was found in JX fermented grains. Meanwhile, JX contained the highest ethyl acetate content but the lowest amount of ethyl lactate. We found that Proteobacteria, Bacteroidetes, and Actinobacteria; Alphaproteobacteria, Actinobacteria, and Bacteroidia; Bacillales, Bacteroidales, and Rhodospirillales; and *Acetobacter*, *Pediococcus*, and *Prevotella_7* in bacterial microorganisms were significantly positively related to four esters and five alcohols, whereas only *Aspergillus* in fungal microorganisms was significantly negatively related to four esters and three alcohols. This study provided evidence of the correlation between the physicochemical properties and the bacteria and fungi contributing to the fermentation process.

## Data Availability Statement

The datasets presented in this study can be found in online repositories. The names of the repository/repositories and accession number(s) can be found below: NCBI, prjna670598 and prjna670601.

## Author Contributions

CL and XG contributed to the experimental design, data analysis, and manuscript writing. GZ, MS, ZJ, and ZY contributed to the experimentation. LL, QZ, TH, XD, and BF contributed to the data interpretation and manuscript preparation. All authors contributed to the article and approved the submitted version.

## Conflict of Interest

The authors declare that the research was conducted in the absence of any commercial or financial relationships that could be construed as a potential conflict of interest.
