# Supplementary material for: Liquor Flavour Is Associated With the Physicochemical Property and Microbial Diversity of Fermented Grains in Waxy and Non-waxy Sorghum (Sorghum bicolor) During Fermentation
Source: Front Microbiol. 2021 Jun 17;12:618458. doi: 10.3389/fmicb.2021.618458 (PMC8247930; doi:10.3389/fmicb.2021.618458)
Supplement: Supplementary file 1 [file Data_Sheet_1.docx]

**Supplementary information:**

**Liquor Flavour Is Associated with the Physicochemical Property and Microbial Diversity of Fermented Grains in Waxy and Non-waxy Sorghum (Sorghum bicolor) During Fermentation**

***Chunjuan Liu^1,2^, Xi******angwei Gong^2^, Guan Zhao^2^, Maw Ni Soe Htet^2^, Zhiyong Jia^3^, Zongke Yan^3^, Lili Liu^3^, Qinghua Zhai^3^, Ting Huang^3^, Xiping Deng^1,*^, Baili Feng^2,*^***

^1^ *College of Life Sciences,* *Northwest A & F University, Yangling, Shaanxi 712100, PR China*

^2^ *College of Agronomy, State Key Laboratory of Crop Stress Biology in Arid Areas/**Northwest A & F University,* *Yangling, Shaanxi 712100, PR China*

^3^ *Shaanxi Xifeng Liquor Company Limited, Fengxiang, Shaanxi 721400, PR China*

**** Corresponding author: Dr. Xiping Deng**

E–mail: dengxp@ms.iswc.ac.cn

Phone: +86–29–87012437; Fax: +86–29–87012210

**** Corresponding author: Dr. Baili Feng**

E–mail: fengbaili@nwsuaf.edu.cn

Phone: +86–29–87082889; Fax: +86–29–87082889

**
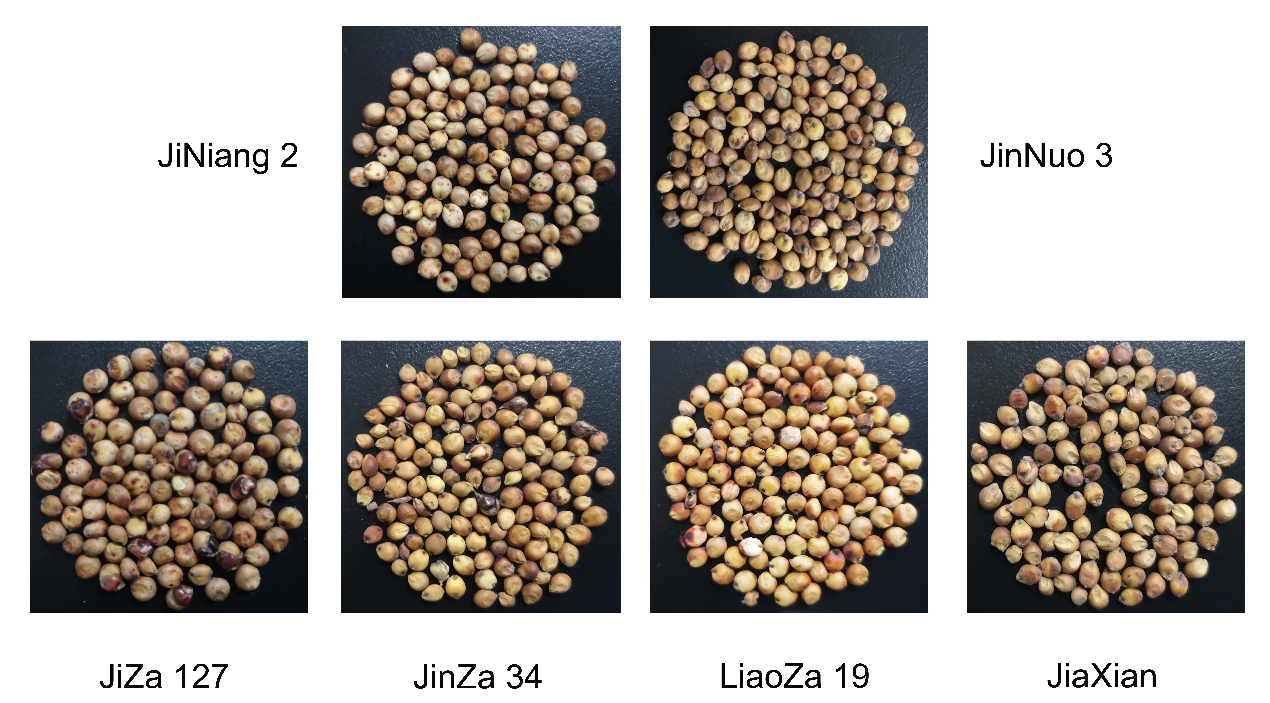
**

**Fig. S1.** Unshelled six sorghum varieties. JiNiang 2 and JinNuo 3 are waxy sorghum and JiZa 127, JinZa 34, LiaoZa 19 and JiaXian sorghum are no-waxy sorghum, respectively.

**Table S1** Quality properties of six sorghum grains.

| Genotype | Variety | Amylose content (%) | Amylopectin content (%) | Total starch content (%) | Protein content (%) | Fat content (%) | Tannin content (%) |
| --- | --- | --- | --- | --- | --- | --- | --- |
| Waxy | JN-2 | 4.70±0.61e | 69.84±0.79a | 74.54±0.19b | 8.38±0.20b | 4.02±0.46a | 1.20±0.04a |
|  | JN-3 | 9.49±1. 41d | 63.14±1.41b | 72.63±0.51d | 8.25±0.13b | 3.78±0.10a | 0.63±0.12c |
| Non-waxy | JZ-127 | 26.90±1.95a | 48.08±1.70e | 74.98±0.37b | 8.84±0.30a | 3.67±0.40a | 0.84±0.09b |
|  | JZ-34 | 17.61±2.10c | 56.21±2.20cd | 73.82±0.29c | 8.31±0.03b | 3.49±0.32b | 0.97±0.09b |
|  | LZ-19 | 22.13±2.02b | 53.11±2.43c | 75.24±0.42b | 8.91±0.11a | 2.98±0.19b | 0.93±0.11b |
|  | JX | 19.47±3.39bc | 57.94±3.73c | 77.42±0.50a | 6.24±0.03c | 3.71±0.26a | 1.22±0.02a |

Six sorghum varieties. JN-2, JN-3, JZ-127, JZ-34, LZ-19, and JX represent JiNiang 2, JinNuo 3, JiZa 127, JinZa 34, LiaoZa 19, and JiaXian sorghum, respectively. Values followed by a different letter within the same column are significantly different at *P* < 0.05.

**Table S2**

Spearman correlation coefficients between the bacterial dominant classification (Phyla, Class, Order and Genera) and the physicochemical properties of fermented grains during the fermentation.

| **Classification** | **Bacterial compositions** | **Temperature** | **Moisture content** | **Starch content** | **Reducing sugar content** | **Acidity** | **Alcohol content** |
| --- | --- | --- | --- | --- | --- | --- | --- |
| **Phyla** | ***Firm*** | **0.735*** | **0.871**** | **0.867**** | **0.895**** | **-0.847**** | -0.700 |
|  | ***Prot*** | **-0.910**** | **-0.780*** | **-0.752*** | **-0.964**** | **0.954**** | 0.566 |
|  | ***Bact*** | -0.446 | **-0.832*** | **-0.803*** | -0.677 | 0.591 | 0.648 |
|  | ***Acti*** | -0.635 | -0.571 | **-0.849**** | **-0.750*** | **0.760*** | **0.875**** |
| **Class** | ***Bacil*** | 0.492 | **0.810*** | **0.873**** | **0.732*** | -0.668 | **-.813*** |
|  | ***Clost*** | -0.017 | -0.537 | -0.681 | -0.320 | 0.245 | 0.788* |
|  | ***Erysi*** | -0.438 | -0.538 | -0.440 | -0.541 | 0.479 | 0.271 |
|  | ***Alpha*** | **-0.920**** | **-0.748*** | **-0.717*** | **-0.954**** | **0.951**** | 0.512 |
|  | ***Gamma*** | -0.301 | -0.676 | -0.680 | -0.542 | 0.477 | **0.818*** |
|  | ***Bacter*** | -0.465 | **-0.829*** | **-0.808*** | -0.685 | 0.600 | 0.657 |
|  | ***Actin*** | -0.635 | -0.566 | **-0.848**** | **-0.748*** | **0.759*** | **0.872**** |
| **Order** | ***Bacill*** | 0.506 | **0.815*** | **0.874**** | 0.743* | -0.680 | **-0.814*** |
|  | ***Lactob*** | **-0.893**** | **-0.731*** | -0.680 | **-0.953**** | **0.965**** | 0.609 |
|  | ***Clostr*** | -0.017 | -0.537 | -0.681 | -0.320 | 0.245 | **0.788*** |
|  | ***Erysip*** | -0.441 | -0.488 | -0.268 | -0.493 | 0.433 | 0.099 |
|  | ***Rhodos*** | **-0.922**** | **-0.733*** | **-0.707*** | **-0.948**** | **0.947**** | 0.493 |
|  | ***Bacter*** | -0.465 | **-0.828*** | **-0.812*** | -0.685 | 0.601 | 0.658 |
| **Genera** | ***Lactobacillus*** | 0.513 | **0.812*** | **0.878**** | **0.748*** | -0.687 | **-0.820*** |
|  | ***Pediococcus*** | **-0.766*** | -0.491 | **-0.708*** | **-0.796*** | **0.844**** | 0.688 |
|  | ***Acetobacter*** | **-0.922**** | **-0.732*** | -0.707 | **-0.948**** | **0.947**** | 0.493 |
|  | ***Prevotella_7*** | -0.446 | **-0.820*** | **-0.809*** | -0.671 | 0.587 | 0.667 |

* Correlation is significant at the 0.05 level. ** Correlation is significant at the 0.01 level.

**Phyla level:** Firmicutes *(Firm),* Proteobacteria *(Prot),* Bacteroidetes *(Bact),* Actinobacteria *(Acti).*

**Class level**: Bacilli *(Bacil),* Clostridia *(Clost),* Erysipelotrichia *(Erysi),* Alphaproteobacteria *(Alpha),* Gammaproteobacteria *(Gamma),* Bacteroidia *(Bacte),* Actinobacteria *(Actin).*

**Order Level**: Bacillales *(Bacill),* Lactobacillales *(Lactob),* Clostridiales *(Clostr),* Erysipelotrichales *(Erysip),* Rhodospirillales *(Rhodos),* Bacteroidales *(Bacter).*

**Table S3**

Spearman correlation coefficients between the fungal dominant classification (Phyla, Class, Order and Genera) and the physicochemical property of fermented grains during the fermentation.

| **Classification** | **Fungal compositions** | **Temperature** | **Moisture content** | **Starch content** | **Reducing sugar content** | **Acidity** | **Alcohol content** |
| --- | --- | --- | --- | --- | --- | --- | --- |
| **Phyla** | ***Asco*** | 0.504 | 0.429 | **0.707*** | 0.497 | -0.460 | -0.231 |
|  | ***Muco*** | -0.513 | -0.428 | -0.704 | -0.502 | 0.465 | 0.229 |
| **Class** | ***Sacch*** | 0.054 | 0.509 | 0.468 | 0.224 | -0.083 | -0.318 |
|  | ***Eurot*** | 0.559 | -0.083 | 0.320 | 0.346 | -0.473 | 0.104 |
|  | ***Mucor*** | -0.509 | -0.421 | -0.700 | -0.495 | 0.462 | 0.220 |
| **Order** | ***Saccha*** | 0.071 | 0.539 | 0.461 | 0.244 | -0.101 | -0.302 |
|  | ***Euroti*** | 0.559 | -0.083 | 0.320 | 0.346 | -0.473 | 0.105 |
|  | ***Mucora*** | -0.509 | -0.421 | -0.700 | -0.495 | 0.462 | 0.220 |
| **Genera** | ***Saccharomyces*** | -0.252 | 0.382 | -0.156 | -0.081 | 0.220 | 0.204 |
|  | ***Candida*** | -0.178 | 0.472 | 0.058 | 0.076 | 0.013 | -0.370 |
|  | ***Naumovozyma*** | 0.366 | -0.009 | 0.043 | 0.155 | -0.195 | 0.309 |
|  | ***Thermoascus*** | 0.391 | -0.290 | 0.192 | 0.150 | -0.277 | 0.175 |
|  | ***Aspergillus*** | **0.805*** | 0.436 | **0.836**** | **0.776*** | **-0.824*** | -0.615 |
|  | ***Rhizopus*** | -0.511 | -0.426 | -0.703 | -0.499 | 0.465 | 0.223 |

* Correlation is significant at the 0.05 level. ** Correlation is significant at the 0.01 level.

**Phyla level:** Ascomycota *(Asco),* Mucoromycota *(Muco).*

**Class level**: Saccharomycetes *(Sacch),* Eurotiomycetes *(Eurot),* Mucoromycetes *(Mucor).*

**Order Level**: Saccharomycetales *(Saccha),* Eurotiales *(Euroti),* Mucorales *(Mucora).*

**Table S4**

Spearman correlation coefficients between the bacterial dominant classification (Phyla, Class, Order and Genera) and the volatile compounds of fermented grains during the fermentation.

| **Classification** | **Bacterial compositions** | Ethyl acetate | Ethyl hexanoate | Ethyl lactate | Ethyl butyrate | N-propanol | N-butanol | [Isobutanol](javascript:;) | [Sec-butyl](javascript:;) [alcohol](javascript:;) | [Isoamylol](javascript:;) | Liquor yield |
| --- | --- | --- | --- | --- | --- | --- | --- | --- | --- | --- | --- |
| **Phyla** | ***Firm*** | **-0.959**** | **-0.951**** | **-0.941**** | **-0.957**** | **-0.787*** | **-0.850**** | **-0.901**** | **-0.936**** | **-0.793*** | -0.540 |
|  | ***Prot*** | **0.931**** | **0.954**** | **0.917**** | **0.969**** | **0.744*** | **0.774*** | **0.835**** | **0.916**** | 0.690 | 0.457 |
|  | ***Bact*** | **0.796*** | **0.779*** | **0.773*** | **0.760*** | 0.658 | **0.732*** | **0.773*** | **0.787*** | **0.721*** | 0.531 |
|  | ***Acti*** | **0.936**** | **0.821*** | **0.927**** | **0.914**** | **0.820*** | **0.940**** | **0.933**** | **0.810*** | **0.829*** | 0.410 |
| **Class** | ***Bacil*** | **-0.896**** | **-0.843**** | **-0.875**** | **-0.854**** | **-0.760*** | **-0.864**** | **-0.887**** | **-0.857**** | **-0.837**** | -0.571 |
|  | ***Clost*** | 0.592 | 0.488 | 0.573 | 0.503 | 0.54 | 0.68 | 0.658 | 0.542 | 0.703 | 0.486 |
|  | ***Erysi*** | 0.619 | 0.576 | 0.599 | 0.605 | 0.53 | 0.564 | 0.562 | 0.502 | 0.593 | 0.625 |
|  | ***Alpha*** | **0.903**** | **0.933**** | **0.893**** | **0.948**** | **0.726*** | **0.740*** | **0.803*** | **0.883**** | 0.656 | 0.426 |
|  | ***Gamma*** | 0.703 | 0.654 | 0.668 | 0.656 | 0.542 | 0.692 | 0.701 | **0.748*** | 0.669 | 0.522 |
|  | ***Bacter*** | **0.803*** | **0.783*** | **0.777*** | **0.769*** | **0.650** | **0.734*** | **0.775*** | **0.798*** | **0.711*** | 0.520 |
|  | ***Actin*** | **0.934**** | **0.818*** | **0.925**** | **0.912**** | **0.818*** | **0.939**** | **0.931**** | **0.806*** | **0.827*** | 0.406 |
| **Order** | ***Bacill*** | **-0.902**** | **-0.852**** | **-0.882**** | **-0.863**** | **-0.764*** | **-0.867**** | **-0.892**** | **-0.865**** | **-0.838**** | -0.571 |
|  | ***Lactob*** | **0.877**** | **0.931**** | **0.867**** | **0.928**** | **0.692** | **0.718*** | **0.785*** | **0.922**** | 0.647 | 0.424 |
|  | ***Clostr*** | 0.592 | 0.488 | 0.573 | 0.503 | 0.540 | 0.680 | 0.658 | 0.542 | 0.703 | 0.486 |
|  | ***Erysip*** | 0.502 | 0.491 | 0.480 | 0.506 | 0.408 | 0.411 | 0.420 | 0.420 | 0.439 | 0.579 |
|  | ***Rhodos*** | **0.892**** | **0.923**** | **0.882**** | **0.939**** | **0.716*** | **0.728*** | **0.791*** | **0.869**** | 0.644 | 0.414 |
|  | ***Bacter*** | **0.804*** | **0.783*** | **0.779*** | **0.770*** | **0.653** | **0.736*** | **0.777*** | **0.797*** | **0.714*** | 0.517 |
| **Genera** | ***Lactobacillus*** | **-0.907**** | **-0.856**** | **-0.887**** | **-0.869**** | **-0.770*** | **-0.873**** | **-0.897**** | **-0.868**** | **-0.841**** | -0.568 |
|  | ***Pediococcus*** | **0.861**** | **0.804*** | **0.868**** | **0.881**** | **0.767*** | **0.816*** | **0.832*** | **0.757*** | 0.701 | 0.299 |
|  | ***Acetobacter*** | **0.892**** | **0.923**** | **0.882**** | **0.939**** | **0.716*** | **0.728*** | **0.790*** | **0.869**** | 0.643 | 0.414 |
|  | ***Prevotella_7*** | **0.797*** | **0.773*** | **0.771*** | **0.760*** | **0.648** | **0.735*** | **0.773*** | **0.790*** | **0.714*** | 0.517 |

* Correlation is significant at the 0.05 level. ** Correlation is significant at the 0.01 level.

**Phyla level:** Firmicutes *(Firm),* Proteobacteria *(Prot),* Bacteroidetes *(Bact),* Actinobacteria *(Acti).*

**Class level**: Bacilli *(Bacil),* Clostridia *(Clost),* Erysipelotrichia *(Erysi),* Alphaproteobacteria *(Alpha),* Gammaproteobacteria *(Gamma),* Bacteroidia *(Bacte),* Actinobacteria *(Actin).*

**Order Level**: Bacillales *(Bacill),* Lactobacillales *(Lactob),* Clostridiales *(Clostr),* Erysipelotrichales *(Erysip),* Rhodospirillales *(Rhodos),* Bacteroidales *(Bacter).*

**Table S5**

Spearman correlation coefficients between the fungal dominant classification (Phyla, Class, Order and Genera) and the volatile compounds of fermented grains during the fermentation.

| **Classification** | **Fungal compositions** | Ethyl acetate | Ethyl hexanoate | Ethyl lactate | Ethyl butyrate | N-propanol | N-butanol | [Isobutanol](javascript:;) | [Sec-butyl](javascript:;) [alcohol](javascript:;) | [Isoamylol](javascript:;) | Liquor yield |
| --- | --- | --- | --- | --- | --- | --- | --- | --- | --- | --- | --- |
| **Phyla** | ***Asco*** | -0.532 | -0.512 | -0.514 | -0.541 | -0.382 | -0.439 | -0.471 | -0.455 | -0.345 | -0.028 |
|  | ***Muco*** | 0.532 | 0.514 | 0.513 | 0.543 | 0.377 | 0.436 | 0.469 | 0.458 | 0.338 | 0.024 |
| **Class** | ***Sacch*** | -0.393 | -0.323 | -0.325 | -0.331 | -0.194 | -0.366 | -0.346 | -0.384 | -0.385 | -0.509 |
|  | ***Eurot*** | -0.184 | -0.246 | -0.249 | -0.270 | -0.253 | -0.104 | -0.172 | -0.093 | 0.035 | 0.598 |
|  | ***Mucor*** | 0.523 | 0.507 | 0.509 | 0.535 | 0.382 | 0.429 | 0.465 | 0.446 | 0.33 | -0.002 |
| **Order** | ***Saccha*** | -0.391 | -0.339 | -0.323 | -0.334 | -0.187 | -0.349 | -0.338 | -0.402 | -0.373 | -0.513 |
|  | ***Euroti*** | -0.184 | -0.246 | -0.248 | -0.270 | -0.252 | -0.103 | -0.172 | -0.093 | 0.035 | 0.598 |
|  | ***Mucora*** | 0.523 | 0.507 | 0.509 | 0.535 | 0.382 | 0.429 | 0.465 | 0.446 | 0.33 | -0.002 |
| **Genera** | ***Saccharomyces*** | 0.139 | 0.037 | 0.176 | 0.167 | 0.209 | 0.223 | 0.180 | -0.034 | 0.088 | -0.365 |
|  | ***Candida*** | -0.289 | -0.205 | -0.285 | -0.196 | -0.358 | -0.363 | -0.354 | -0.247 | -0.408 | -0.583 |
|  | ***Naumovozyma*** | -0.057 | -0.07 | -0.081 | -0.105 | -0.073 | 0.036 | -0.009 | 0.037 | 0.171 | 0.348 |
|  | ***Thermoascus*** | -0.054 | -0.056 | -0.111 | -0.122 | -0.142 | -0.032 | -0.056 | 0.096 | 0.102 | 0.606 |
|  | ***Aspergillus*** | **-0.809*** | **-0.769*** | **-0.808*** | **-0.844**** | -0.658 | **-0.732*** | **-0.759*** | **-0.724*** | -0.577 | -0.060 |
|  | ***Rhizopus*** | 0.527 | 0.511 | 0.512 | 0.538 | 0.385 | 0.432 | 0.468 | 0.451 | 0.333 | 0.002 |

* Correlation is significant at the 0.05 level. ** Correlation is significant at the 0.01 level.

**Phyla level:** Ascomycota *(Asco),* Mucoromycota *(Muco).*

**Class level**: Saccharomycetes *(Sacch),* Eurotiomycetes *(Eurot),* Mucoromycetes *(Mucor).*

**Order Level**: Saccharomycetales *(Saccha),* Eurotiales *(Euroti),* Mucorales *(Mucora).*
